# Narratives of contingency as historical evidence for philosophical arguments of contingency: pathway decisions in the early development of molecular genetics

**DOI:** 10.1007/s40656-026-00738-1

**Published:** 2026-06-10

**Authors:** Wessel de Cock

**Affiliations:** Humboldt University in Berlin, Berlin, Germany

**Keywords:** HPS, Development of science, Narratives of contingency, Contingency / Inevitability debate, Molecular biology

## Abstract

This article argues that philosophical arguments of contingency in the development of science can be advanced by starting from historical evidence produced through the framework of narratives of contingency. Narratives of contingency are conceptualized both as prospective narratives constructed by historical actors to navigate decisions at what they perceived as fork moments in the present and as an analytical framework for systematically reconstructing such decision-making processes from historical sources.

The argument is developed in three steps. First, the article outlines three constraints that historical case studies must explicitly address in order to function as evidence for philosophical arguments of contingency and alternative pathway possibility. These are the identification of a juncture moment in a situated trajectory, an explanatory factor linking actors’ decisions to the initiation of path-dependent sequences, and an explicit specification of analytical level and scale. Second, narratives of contingency are introduced as a historical framework that enables the systematic description and explanation of pathway decision-making processes, particularly in contexts of institutional deliberation where alternative courses of action were explicitly articulated. Third, the framework is illustrated through a case study of institutional decision-making in the early development of molecular genetics in Germany (1958–1960).

## Narratives of contingency as a type of historical evidence for philosophical arguments of contingency in the development of science

Thomas Kuhn's *The Structure of Scientific Revolutions* of 1962 has been recognized concurrently as the starting point and pinnacle of an integrated History and Philosophy of Science (HPS) (Golinski, 2024). Philosophers and historians of science alike took inspiration from *Structure*, but where it made philosophers consider how historical cases can provide an empirical foundation for philosophical theorization about the development of science, it moved, or at least reinforced, historians’ attention for local shifts in scientific practices (Kuukkanen, 2016; Mauskopf & Schmaltz, 2011; J. H. Zammito, 2011). Combined with a concurrent divergence through institutionalization, calls in the 1990s by interdisciplinary-minded scholars for an integrated HPS had little effect (Schickore, 2011). Whilst recent years have witnessed a resurgence of philosophical interest in historical case studies to advance problems such as contingency or inevitability (C/I) in the development of science, historians of science have, with a few notable exceptions (Radick, 2008), remained remarkably absent as participants.

Although the C/I problem was initially raised by prominent Canadian philosopher Ian Hacking at the height of the Science Wars in the 1990s (Hacking, 1999), its systematic development only began in the late 2000s (Soler, 2008). Since then, philosophers have largely come to agree that a productive C/I debate is not so much about the extent to which the outcomes of science are either contingent or inevitable but rather, about which elements in situated cases were causally dependent for particular developments and if and how alternative pathways were possible (Kinzel, 2015b; Martin, 2013; Soler et al., 2016). This makes the problem not only connected to other key debates in philosophy of science such as scientific realism or the internal/external debate, but, as Lena Soler demonstrated, also an important “autonomous question”, whose answering historians of science can contribute to and benefit from (Soler et al., 2016, p. 14). As Kinzel has clarified, philosophical arguments are namely mainly hindered by a combination of lack of understanding of how historical evidence is produced and an incapacity to conceive of types of historical evidence that could act as a “neutral arbiter in a philosophical conflict” (Kinzel, 2015a, p. 21).

The aim of this article is to introduce an analytical framework for a specific type of evidence, narratives of contingency, to the predominantly philosophical-normative C/I debate. As De Cock and Reinhardt have proposed, narratives of contingency can be thought of as a type of prospective narrative that historical actors construct to navigate individual or community decisions at what they perceive as a fork moment in the present (de Cock & Reinhardt, 2024). Contingency is understood here from the perspective of the actors themselves: the conditions in the past or anticipated future that they identify as causally consequential for the direction of a trajectory, and that they recognize as capable of having been or being otherwise. This differs from contingency as a bifurcation point in an experimental sequence at which a decision must be made without sufficient grounds to guide it, and from contingency as a radically unforeseen finding arising from within a research trajectory that redirects its future orientation—both genuine forms of scientific contingency that fall outside the present scope.

This article proposes that narratives of contingency can serve as an analytical framework (A. Currie & Walsh, 2019; Virmajoki, 2023b) for the systematic description and explanation of prospective decision-making processes in which a number of possible courses of action were outlined, compared, put into motion, or rather disregarded.^1^ Accordingly, I focus on meso-level institutional decision-making in the life sciences, specifically the development of the discipline of genetics in the late 1950s and early 1960s. Historical case studies constructed through this framework can then allow philosophical arguments to move away from increasingly criticized speculative counterfactuals (Dagg, 2019; Tambolo, 2016, 2020; Virmajoki, 2022), to historically-initiated arguments for contingency in the developmental direction of science (McGhee, 2016).^2^

### The impasse in the philosophical-normative C/I debate

It is now broadly recognized that both philosophical claims of inevitability and contingency—that science would always eventually “converge” on a outcome roughly similar to current science, or that alternative paths are fundamentally possible—are conditional rather than absolute statements (Beatty, 2006; Sankey, 2008, pp. 260–261).^3^ Philosophers of science have accordingly initiated a move away from Ian Hacking’s original linear scale towards taxonomies of possible contingency or inevitability arguments (Aylward, 2019; Kinzel, 2015b; Martin, 2013). After all, as Jeroen Bouterse recently emphasized, the point of an argument is to be “interesting and not trivial”, for example, by revealing certain theoretical philosophical commitments, or by making explicit claims about contingency in the narratives of historians of science (Bouterse, 2022, p. 28). For instance, Joseph Martin has observed that philosophical arguments of contingency typically place emphasis on certain elements of science—ontological, methodological, interpretive, or social factors like a scientific community—thus claiming “causal dependency” between an explanatory factor and a particular outcome (Martin, 2013, p. 921). Kinzel similarly showed that normative C/I arguments typically consists of answers to four interrelated questions about alternative pathways: “(how) are they possible, what types are we talking about, how should they be assessed, and how different are they from actual science” (Kinzel, 2015b, p. 22)?

This shows that while C/I arguments are no longer seen as positions on a linear scale, the so-called success criteria originally formulated by Hacking—that an alleged alternative pathway must be comparably successful to our current science—continues to be the main parameter in philosophical contributions to the problem (Bouterse, 2022; Tambolo, 2020). This is understandable, seeing that philosophers of science habitually depart from norms of present’ day science and because, as Bouterse points out, “at first sight the success criterion is there for a reason, if we allow less successful alternatives to enter, contingentism seems to become trivially true” (Bouterse, 2022, p. 28). Nevertheless, the “default position” that requires proof of equivalent success from any contingency argument, something Hacking characterized as “Put Up or Shut Up”, has prevented the C/I problem from evolving into a productive debate (Aylward, 2019; Soler et al., 2016). As Soler has explained, even if contingentists “put up” historically instantiated alternatives, inevitabilists can dismiss these by constantly expanding the demand for evidence of its long-term epistemological legitimacy (Soler et al., 2016, p. 25). This, arguably inevitably, has largely redirected philosophical arguments from historically plausible evidence towards speculative counterfactual trajectories, which Kinzel has called “meaningless tautologies”, and Kidd describes as “hubris” (Kidd, 2016; Kinzel, 2015b, p. 20).

Accordingly, Bouterse has recently convincingly argued for a historical (re)formulation of the C/I problem where “what defines a genuine alternative” is not an alternative pathway’s eventual similarity to current science but rather “its ‘branching off’ from the same preceding state” (Bouterse, 2022, p. 30). The targets for arguments of contingency or inevitability would then revolve around how and why there were (or were not) alternative pathways that, at a particular stage in a developmental trajectory of science, could plausibly have been put into motion and developed. Bouterse's “extreme conclusion”—that philosophers should concentrate on the systematic and normative aspects of the problem and historians on the descriptive historical elements—is nevertheless unwarranted (Bouterse, 2022, p. 28).^4^ After all, the current impasse in the philosophical debate shows that this historical descriptive evidence is necessary for any productive normative philosophical argument. At the same time, it is both possible and necessary for historians of science to consider the norms of contemporary science when describing the viability of alternative pathways in what Loison has termed “critical presentism” (Loison, 2016, p. 36).^5^ Accordingly, the next step is to make explicit the constraints a historical case study must meet in order to function as evidence in normative philosophical arguments.

## Constraints for historical evidence to be viable for philosophical arguments of contingency and alternative pathway possibility

Philosophers of science have long envisioned the history of science to provide case studies that could serve as empirical data for philosophical generalizations (Chang, 2011). This “confrontational model” has been shown to face the insoluble dilemma of starting either by linking a philosophical concept to a historical case, risking bias and cherry-picking, or vice versa with a single historical case and face the challenge of generalizability (Pitt, 2001). As solution, scholars like Raphael Scholl have proposed to make this interaction an “iterative process” in which, as Bolinska and Martin summarize, “abstract theory is refined upon repeated contact with concrete historical cases” (Bolinska & Martin, 2020, 2021; Schickore, 2018; Scholl, 2018). Kinzel has however pointed out that these proposals still depart from an “oversimplified idea of historical evidence” that ignores or outright denies the theory-ladenness and narrative nature of history (Kinzel, 2015a, p. 8).^6^ Nevertheless, Bolinska and Martin have shown that this does not disqualify historical case studies from being used as evidence in philosophical arguments in the C/I debate (Bolinska & Martin, 2021).^7^ They argue that, in addition to standard practices of historiographic “methodological caution” such as source critique, this would require historians to establish an explicit “canonical connection” between their explanatory factors and the particular philosophical problem they aim to contribute to (Bolinska & Martin, 2021, p. 15).

This raises the question of what the main constraints are that historical case studies should fulfil in order to function as evidence in philosophical arguments about contingency.^8^ These constraints can be identified by taking into account the above clarifications of philosophers and by looking at the few historically-instantiated case studies that have been broadly accepted as evidence by philosophers in the C/I debate. A primary example is the work of philosopher and historian of science Gregory Radick on early twentieth century genetics (Radick, 2008, 2023). Furthermore, efforts to define contingency and pathway dependency in the philosophy of history, sociology and political science will be drawn upon to define three constraints that such case studies should systematically and explicitly address.

First, a case study should explain why a particular stage in a situated developmental trajectory of science represents a critical juncture where multiple pathways could have plausibly been put into motion. As political scientists Capoccia and Kelemen have outlined, critical junctures are “relatively short periods of time” during which certain structural constrains are “significantly relaxed”, creating a “substantially heightened probability that agents' choices will affect the outcome of interest” (Capoccia & Kelemen, 2007, p. 348). Critical junctures are consequently a dual analytical and actor category (Poskett, 2024; Soifer, 2012; Steinmetz, 2008).^9^ This means that an assertion of a critical juncture must, on the one hand, illustrate the loosening of structural constraints, which can range from “internal” material, technological or mental-cognitive, to “external” political, social and cultural conditions (Galison, 1995, p. 15).^10^ On the other hand, it has to demonstrate that key decision-makers were uncertain about the future development of the trajectory to which their possible actions in the present were tied. Critical junctures can accordingly turn into a fork-moment when, as historical sociologist James Mahoney notes, “a particular option is adopted among two or more alternatives” (Mahoney, 2000).

Second, the main explanatory factor of a case study must represent a causal factor setting into motion an identified (alternative) pathway. Following contributions by historians and philosophers of science like Yemima Ben-Menahem, John Williamson, Veli Virmajoki and Adrian Currie, causal factors in historical narratives are primarily understood as “difference makers” (Ben-Menahem, 1997; A. M. Currie, 2014; Virmajoki, 2020, 2023a). That is to say, as John Williamson has summarized, that causality is understood as a “feature of the way reality is represented” rather than as a “feature of agent-independent reality itself” (Williamson, 2013, p. 268). As Rob Inkpen and Derek Turner have argued, causal explanatory factors of contingency in historical narratives are therefore necessarily linked to the (possible) actions of historical actors. After all, when historians make a (usually implicit) claim that a certain historical outcome was inevitable, this implies that “those outcomes are insensitive to things that human actors do”. Contingency, on the other hand, means that initial “small actions can make a big difference to later results” (Inkpen & Turner, 2012, p. 2). This means that this historical explanatory factor is necessarily linked to a claim that a particular action would set into motion a certain path dependency.

Sociologists have differentiated between two types of path dependency: “reactive” dependency, where each new event in the sequence is a direct consequence of the initial contingent event, and the more common “self-reinforcing sequences” (Goldstein et al., 2023; Sydow et al., 2009). As Vergne and Durand clarified, this reinforcement mechanism is often not so much “increasing returns”, but rather, a process where “the relative attractiveness of alternatives” decreases and “alternative paths are selected out” (Vergne & Durand, 2010). In the context of science, this occurs for example when scientists or research groups continue with a certain experimental setup, not because this delivered the productivity they had initially envisioned, but rather because changes are considered too costly or risky (Böschen et al., 2024, p. 95). Subsequently, pathways can be retrospectively argued to have “locked-in” and reached a temporal “state of equilibrium with a very low potential for endogenous change” (Vergne & Durand, 2010, p. 743). A claim of contingent path dependency accordingly can be argued to consist of two components. On the one hand, the argument that a pathway was contingently entered because alternatives were plausibly possible. On the other hand, it has to include an explanatory factor of why there would occur a temporal “lock-in” on this particular direction over a period of time or towards some outcome.

Thirdly, this highlights that historical evidence of contingency and the possibility of alternative pathways is not only descriptively sensitive, as Ben-Menahem emphasized, but is also a theory-laden process with regard to scale (Ben-Menahem, 1997). After all, as Inkpen and Turner summarize, in order to plausibly argue that specific actions would set into motion an (alternative) pathway with a certain path dependency, it matters how the boundaries of this system are drawn in the first place (Inkpen & Turner, 2012, p. 2). Do these actions solely relate to the micro-experimental level, the meso-institutional or also impact on the macro-field or discipline level? And, how do these necessarily situated cases relate to the essentially global development of scientific knowledge (Nappi, 2013)?^11^

Although these three constraints are often implicitly and partially present in historical narratives, for case studies to function as evidence for philosophical arguments of contingency they need to be explicitly addressed and systematically organized. This becomes evident when looking at the remarkably broadly accepted case study and argument for contingency in the development of the “science of inheritance” by Gregory Radick (Comfort, 2023; Radick, 2024).^12^ Firstly, Radick explicitly identifies a juncture moment in this trajectory with the debate between Weldon and Bateson about the “interpretation of Mendel's experiments” in Great Britain of the early 1900s. He argues that the “swift establishment of a new science based on the gene” occurred because this particular debate “went as it did” (Radick, 2023, p. 4). In describing this local debate he emphasizes to “take seriously what those involved took seriously” (Radick, 2023, p. 280), noting that both Weldon’s course of action was shaped by his “vision for the future of biology” and Bateson too was “fighting as if nothing less than the future of biology was at stake” (Radick, 2023, pp. 7, 179). Thus, he implies that this was also considered by actors as a genuine juncture moment. Secondly, Radick’s explanatory factor explicitly focusses on “human- scale causes and reasons” such as “the doings and decisions of individual men and women”, “choices Bateson made, and why he made them”, and “decisions that turned out to be highly consequential” rather than on “summoning-forth explanations” (Radick, 2023, pp. 14, 169, 280, 191). Thirdly, Radick is explicit about how this essentially local meso-institutional level debate connects to broader developments on the micro-experimental and macro-disciplinary level (Radick, 2023, pp. 2, 295–298). Thus, when making his argument for contingency in the broader trajectory of genetics, he focusses on explaining why Bateson's vision of genetics expanded so rapidly after Weldon's death. Although he lists a number of underlying factors such as that most scientists as any “moderately ambitious people [..] tend to go with the flow”, his main explanatory factor is Weldon’s emphasis on a “combination of teachable principles, tractable problems, and technological promise” (Radick, 2023, p. 279).

## Narratives of contingency: an historical framework for case studies of pathway decision-making processes

The preceding section has identified three constraints that historical case studies must explicitly address if they are to function as evidence in normative philosophical arguments about contingency and alternative pathway possibility in the development of science. Such case studies must (i) locate a juncture moment in a situated trajectory, (ii) articulate an explanatory factor that links actors’ actions to the (possible) initiation of a path dependency, and (iii) make explicit the level and scale on which the development of an (alternative) pathway is being assessed. In what follows, I introduce narratives of contingency and clarify how it can be employed as an historical framework of analysis that explicitly fulfills these constraints.

As developed by De Cock and Reinhardt, narratives of contingency designate anticipatory narratives that scientific decision-makers construct at what they perceive as juncture moments in the present (de Cock & Reinhardt, 2024). Conceptually, the construction of such narratives draws on what Reinhart Koselleck described as the anthropological categories of human temporal experience: the space of experience (the present past) and the horizon of expectation (the present future) (Koselleck, 1979, pp. 352–360; J. Zammito, 2015).^13^ By selectively emplotting past experiences and anticipated future conditions around a node in the present, these narratives allow actors to articulate, compare, and evaluate alternative courses of action.

The central claim of this article is that narratives of contingency can be employed as an analytical framework that fulfills the constraints identified above and thereby produces historical evidence usable for normative philosophical arguments in the contingency/inevitability (C/I) debate. For example, such analysis may reveal that decision-makers outlined and compared two viable directions and ultimately put one into motion by mobilizing particular past experiences to justify specific anticipated future conditions. This can then ground a normative philosophical argument that the subsequent directionality of the trajectory was contingent upon those decisions and their enabling conditions, especially where these can be plausibly linked to self-reinforcing dynamics that reduced the feasibility or attractiveness of alternative directions over time.

The theoretical validity of this focus on the construction and employment of anticipatory narratives in pathway decision-making processes is supported by two overlapping strands of recent research across history and philosophy of science, science studies, and cognitive and futures-oriented studies. First, a range of studies has shown that narratives are central to scientific reasoning, coordination, and explanation, including in the organization and sequencing of experimental work and in strategic project planning (Beatty, 2016; Carrier et al., 2021; Goodson & Gill, 2011; Milojević & Inayatullah, 2015; Morgan & Wise, 2017; Morgan et al., 2022). Second, research in social and cognitive science has suggested that narrative construction is a key tool through which actors relate past experience to anticipated futures and thereby make deliberation, choice, and action possible (Beach, 2009; Constantino & Weber, 2021; Johnson et al., 2023; Milojević & Inayatullah, 2015; Tuckett & Nikolic, 2017).

Thus, as an analytical framework, narratives of contingency can meet the three constraints identified above for producing historical evidence usable in philosophical arguments in the contingency/inevitability debate.

First, narratives of contingency help fulfil the juncture constraint by showing when and why a period was experienced as one of heightened uncertainty, in which multiple directions appeared viable. The framework is especially well suited to identifying fork moments, where actors understood each choice as carrying path-dependent consequences. Examples on the meso-level are the institutionalization of a research direction through appointments, the demarcation of a field through editorial policy, or the prioritization of certain lines of investigation through strategic planning and funding application.

Second, narratives of contingency operationalize the explanatory-factor constraint by reconstructing which (possible) conditions actors treated as decisive when choosing between competing directions at a perceived fork moment. It makes visible contemporary epistemic assessments such as the perceived strengths and limits of techniques, model organisms, and problem-choices and highlight how and why these intertwined with prospective evaluations of experimental feasibility, institutional capacity, scientific productivity, as well as “external” conditions such as funding regimes, political support, and societal relevance. Thus, this framework can identify an historically plausible explanation of *why* one option was selected and how that choice could plausibly initiate or reinforce a path dependency: through appointments, resource allocations, demarcation decisions, and the progressive selection-out of alternatives as their relative attractiveness or feasibility decreased over time. Recent lines of sociological investigation support the plausibility of this explanatory factor by showing that research groups and institutions routinely navigate uncertainty by weighing future risk and reward, balancing “productive tradition” against “occasional gambles for extraordinary impact,” and often favoring familiar lines over higher-risk options that might promise major contributions but also a higher probability of being “unproductive” (Besancenot & Vranceanu, 2014; Foster et al., 2015, p. 8; Liu et al., 2024).

Third, narratives of contingency help satisfy the level and scale constraint because they can be traced to specific situated pathway decision-making. Consequently, this allows for explicitness about *where* the initial pathway decision is located; on the micro-experimental, meso-institutional or macro-policy-level. At the same time, it provides an explicit way of moving from local cases to broader, field-level claims. By comparing decision-making processes across sites, it is possible to show which conditions are site-specific and which circulate across a wider community (Hilgartner, 2015; Jasanoff & Kim, 2015; Knorr-Cetina, 1999) This allows for the cautious form of generalization that is necessary for normative philosophical C/I arguments. For example, local case studies can be compared for recurring narrative structures, decision logics, and cited prospective conditions or past experiences. Such patterns can then be used as evidence to show how directionality in a field becomes enacted and subsequently stabilized through multiple, and occasionally intertwined, decision-making processes.

## Pathway decision-making processes at the emergence of molecular genetics in Germany (1955–1965)

Having delineated narratives of contingency as a historical framework, this section demonstrates in two steps how it can produce historical case studies that subsequently can be used as evidence in philosophical arguments about contingency and alternative pathway possibility. First, I apply the framework to my archival research on the debates over the future direction of genetics in West Germany in the late 1950s and early 1960s. This will reveal (i) how a juncture moment turned into a fork moment for decision-makers when they had to make institutional pathway decisions (ii) how competing narratives of contingency structured these discussions and (iii) how these subsequently initiated a meso-level path dependency as one direction was institutionalized and another disregarded. Secondly, I contextualize this local case within the broader history of early molecular genetics (1955–1965) to demonstrate the occurrence of similar processes across different locations, which supports more cautious claims at the macro-field level that help explain the direction of development of genetics.

The rise of molecular approaches in investigative trajectories ranging from virus and bacterial genetics to biochemistry and biophysics in the mid-1950s, particularly in the United States, prompted decision-makers worldwide to discuss the possible implications for their institutions. In Germany, these discussions are traceable across correspondence between key decision-makers active in the Max Planck Society and editorial boards of a variety of biology and genetics journals published by the Verlagshaus Springer of Ferdinand Springer (junior). While these actors generally acknowledged that molecular approaches and techniques held some potential for certain lines of investigation in biology, they had different assessments on whether they, as publisher Heinz Götze noted to Melchers in 1959, would “gain significance outside of genetic and virus research” in the long-term.^14^ At the same time, as head of the medical-biological section of the MPS Gunther Lehmann observed, stakeholders were increasingly questioning whether the”practice of mammalian genetics can still be fruitful today”.^15^ Having experienced significant upheavals throughout their careers, these actors therefore approached this as a genuine juncture moment where multiple developmental directions for the discipline of genetics were possible. These upheavals ranged from the rapid expansion of genetics into various branches in the 1920s, which often marked the beginning of their scientific careers, to the broad perception that German biological research had lost its pioneering role in the discipline due to the war. On top of that, as many historians of molecular biology have also observed, this period was globally and interconnectedly experienced as one of wide-ranging change in the scientific, technological, societal and political spheres (Abir-Am, 1992, pp. 166–167).

This juncture moment turned into a fork moment when institutional decisions had to be made that decision-makers perceived as carrying substantial path-dependent consequences, in particular appointment processes. In Germany, these processes took place over roughly 2 years, between 1958 and 1960. In 1958, it became clear that a replacement had to be selected for the directorship of the Max-Planck Institute for Vergleichende Erbbiologie und Erbpathologie in Berlin after its director, Hans Nachtsheim, was announced to retire. In parallel, in 1959, Georg Melchers, the editor in chief of the *Zeitschrift für induktive Abstammungs- und Vererbungslehre*, proposed appointing new editors and broadening, or even redirecting, the journal’s scope to “molecular genetics.” Because these debates largely involved the same small group of decision-makers and overlapping narratives, and for reasons of analytical and argumentative clarity, I focus primarily on the discussions about the appointment of a new directorship at the Berlin genetics institute.

There are several reasons why involved stakeholders within the commission perceived director appointments a fork moment where their collective decision would set into motion a new trajectory, positive or negative, for their own careers, institutes and genetics research in Germany. Firstly, this had to do with the important status of the MPS, which was considered as Germany’s leading organization for basic research, encompassing a wide range of largely autonomous institutes across disciplines. Secondly, the MPS administration was characterized by the Harnack Principle in which institute directors were given lifetime positions with near-unlimited power to shape the institutional direction (Renn et al., 2024). Thirdly, these institutes were expected, both internally and externally, to set the direction in basic research in Germany and provide contributions that could define the direction of the discipline globally. In this context, the commission was tasked not merely with filling a vacancy, but with anticipating the future development of genetics and recommending directors whose investigative pathways held the promise to define that development and secure the organization success, ideally in the form of a Nobel Prize.

To meet the second constraint, I now reconstruct how decision-makers linked candidates to potential lines of investigation by constructing narratives linking specific past experiences to anticipated future conditions. Two competing narratives of contingency can be identified that structured the subsequent pathway decision-making processes by making a candidate and direction appear more promising, risky, or viable than others.

The first narrative framed the future success of the institute as contingent upon appointing bacteria geneticist Fritz Kaudewitz as sole director. It was advanced primarily, in various degrees of radicality, by biochemist and Nobel Prize Winner Otto Warburg (MPI Cell Physiology) and Feodor Lynen (MPI Cell Chemistry), who would win a Nobel Prize in 1964. Rather than emphasizing Kaudewitz’s specific program or past achievements, their narrative suggested that classical mammalian and human genetics was not capable of generating fundamental new discoveries in the future. For example, Warburg repeatedly emphasized that mammalian genetics in the “past 30 years had not brought anything fundamentally new and has become sterile” and therefore “belonged in the health offices” as an applied science.^16^ While Lynen admitted that mammalian genetics might have some fundamental questions left on its horizon, he highlighted Nobel prize nominations in recent years as proof that “the focus of genetic research today is undoubtedly in the field of bacterial genetics”.^17^

Their narrative heavily drew upon comparisons to the collective space of experience of the pre-war success of the Kaiser Wilhelm Society, the predecessor of the MPS. According to Lynen and Warburg, the KWS had proven that “truly fundamental discoveries”, or “breakthroughs” defining for a field or discipline, were contingent upon an institute where “all time was dedicated to experiments” and led by a “young man in his most fertile creative period”. In addition, Lynen suggested that the alternative possibility of a “coordination of mammalian geneticists and bacterial geneticists under one roof” was unlikely to succeed because past examples had shown that “cooperation between great and creative researchers is rarely achievable”. Specifically, he referred to the creation in 1927 of the Kaiser Wilhelm Institute for Medical Research in Heidelberg “where it was firmly believed that bringing chemistry, physiology, and physics together under one roof would establish a kind of ‘teamwork’ in frontier areas of medicine”. Nevertheless, as he emphasized, this did not take place, and if the objective is to “find a man who can make fundamental discoveries”, this was simply “much more easily [found] among bacterial geneticists than among animal geneticists”.

Although Lynen, and particularly Warburg, also narrated future scientific conditions for why Kaudewitz’ direction would be the right one, for instance that “modern genetics is nothing else than chemistry or physics of nucleic acids”, the main point of their narrative was that the new model organisms would allow for an “return to the principles of the KWG”.^18^ Thus, one of the main arguments of Warburg was that bacteria were “much easier to handle and require less personnel and material compared to human or animal genetics”.^19^ Similarly, Warburg warned against the possible “acceptance of two million from the Atomic Ministry” as this would make the “institute to too large and require too much administrative work”.^20^ To summarize, Warburg and Lynen argued that the institute’s future capacity to shape the field was contingent upon restoring an institutional arrangement that had proven successful in the past, and they presented bacterial genetics as the most plausible means to realize that form.

The second narrative, by contrast, argued that a “big win” for the institute was contingent upon integrating bacterial genetics with classical mammalian and human genetics in a productive exchange “under one roof”.^21^ This narrative was pushed by the majority of the commission, including its head, Gunther Lehmann, and successive MPS presidents, Otto Hahn and Adolf Butenandt. While most agreed with Lynen and Warburg that there was a “considerable gap between the rapidly advancing field of bacterial genetics and the genetics of higher animals” that in recent years “has fallen far behind”, they, like Lehmann, emphasized that “at some point this deficit has to made up”, making a collaboration “especially interesting”.^22^

Their narrative argued that success of genetic research was contingent upon ensuring political interest and thereby funding and that for this reason classical mammalian and human genetics would continue to be highly relevant. The influential director of medical research at the MPS, Karl Thomas, for instance, emphasized that the German Science Council (Wissenschaftsrat) had encouraged the creation of new departments for classical human genetic research. His narrative further anticipated the future expansion of the welfare state and the resulting lack of “timely elimination or at least suppression of genetically undesirable offspring” to the rise of significant political interest in classical mammalian and human genetics.^23^ Thus, he effectively suggested that the continuation of mammalian and human genetics was contingent upon the political interest in eugenics. Furthermore, he and others in the commission repeatedly highlighted the growing importance of nuclear energy, or even a possible nuclear war, as future contingent factors that would ensure state interest and funding for heredity research. The molecular techniques of Kaudewitz's “bacterial genetics” would then, as Lehmann narrated, provide classical genetics the “much-needed impetus that is highly desirable for a variety of reasons”.^24^ They also highlighted analogous directions that they anticipated to be institutionalized elsewhere. For example, Lehmann noted that “something similar for the field of botany” was being organized for the new genetics institute in Cologne, which was being conceptualized with the support of the prominent phage geneticist Max Delbrück. To summarize, the integration narrative presented future success as contingent upon combining political relevance and funding for mammalian genetics with an epistemic “impetus” from bacterial genetics and therefore treated an integration “under one roof” as the direction contingent for success.

This integration narrative initially seemed to prevail as the commission proposed a dual appointment of Kaudewitz and mammalian and human geneticist Hans Grüneberg, who had fled Germany in the 1930s for Great Britain when the Nazis came to power. Nevertheless, the integration pathway depended entirely on the availability of Grüneberg who was repeatedly presented as the only German-speaking “first class researcher” in classical mammalian genetics. This narrowing was reinforced by the fact that Kaudewitz, too, had few credible competitors, being presented as the only available young bacterial geneticists in West Germany. When Grüneberg by mid-1960 withdrew his candidacy, possibly after an apparent incident of antisemitism at the presentation of his research plans in Berlin, the integration option was treated as no longer implementable.^25^ In other words, the withdrawal did not replace the prior pathway decision-making process; it resolved it by collapsing the integration pathway just before it was enacted, thereby clearing the way for Kaudewitz’s sole directorship. In the immediate aftermath, Kaudewitz rapidly began dismantling the institute’s mammalian and human genetics pathways, including by forcing out younger researchers such as Friedrich Vogel.^26^

Although Kaudewitz himself would leave the institute for the University of Munich in 1962 due to suddenly worsening political situation in Berlin, there is no evidence that his departure reopened the discussion about the epistemic orientation. By 1964, the institute was officially renamed “molecular genetics” and placed under the directorship of three young scientists whose specializations aligned with research directions that were being institutionalized at prominent sites internationally. The institute’s focus would remain largely non-mammalian until 1994, when the appointment of new directors Hans-Hilger Ropers (born 1943) and Hans Lehrach (born 1946) marked a renewed turn toward human genetics, now pursued with genomic experimental approaches.

Firstly, this case study provides evidence to support the argument that the institutional direction of the Berlin institute, and the marginalization of classical mammalian and human genetics there, was contingent upon the deliberative pathway decision-making processes of a select group of institutional stakeholders. These discussions were structured by two narratives of contingency linking potential candidates and directions to past experiences and anticipated future conditions. Because the majority of decision-makers was convinced of the narrative that the future development of genetic research would be contingent upon securing political support, classical mammalian and human genetics were broadly envisioned to remain an important direction in genetics. Moreover, like Charlotte Auerbach cautioned Melchers against “putting all eggs in one basket”, many decision-makers at that point in time seemed reluctant to commit exclusively to new model organisms such as phages and bacteria.^27^ This also helps explain why these actors’ evaluations changed so rapidly, and why the molecularization was embraced so broadly once, in the early 1960s, national governments in Europe began making significant funding available for the institutionalization of molecular approaches in order to “catch up” with apparent advances elsewhere (Strasser, 2002).

Secondly, although this integration ultimately did not occur at the MPS, it nevertheless represents a historically plausible alternative pathway that could have been put into motion. Moreover, it can be argued that this alternative pathway contains a certain epistemological legitimacy and, consequently, a plausible path-dependency that extends beyond the mere fact that these directors would have been in control over the institute until retirement. After all, Grüneberg’s medically driven research on gene mutation in mice was identified at the time as being of high potential, as is testified by the funding he received from the British Medical Council and the Rockefeller Foundation and the fact that he was made a Fellow of the Royal Society (FRS) in 1956 (Berry, 1983; Lewis et al., 1982). In addition, the research program of Grüneberg, who has retrospectively been praised as one of the “fathers of mouse genetics”, in productive engagement with Kaudewitz bacteria genetics, represents an alternative pathway in which molecular methods would be applied to (classical, phenotype-driven) mammalian and human genetics roughly a decade earlier than they actually did (Paigen, 2003; Suárez-Díaz Edna & García-Deister Vivette, 2015).

### Evidence for normative arguments about contingent directionality. From the local to the global

Thus far, the framework of narratives of contingency has produced evidence for normative philosophical arguments about contingency and alternative pathway possibility at the local meso-level. To support historically sound field-level arguments about contingency and alternative pathway possibility, comparable processes must be identified across different institutional settings. In what follows, I advance a normative-philosophical contingency claim about directionality: the rapid narrowing from integration options that many actors still treated as viable in the 1950s to a disciplinary “molecular” trajectory in the early 1960s was causally sensitive to how decision-makers abruptly re-narrated the future of genetics. In particular, I argue that anticipated developments elsewhere were taken up as new emplotments that underlined the inevitability of a “molecular” direction and helped select out integration pathways. Read through the framework of narratives of contingency, historiographical case studies can therefore function as evidence for recurring (i) juncture experiences, (ii) integration alternatives, and (iii) a mechanism through which meso-level lock-in occurred across different sites.

Firstly, the historiography of molecular genetics shows that the period between the late 1950s and mid 1960s was broadly perceived and experienced as a juncture moment, in which institutional actors confronted an unusually contested sense of possible futures for genetics. As Pnina Abir-Am has shown, the historiography consists of local institutional case studies documenting “series of skirmishes” between a remarkably small yet diverse group of proponents for molecular approaches and a majority of hesitant or opposed decision-makers in university administrations, peer review commissions, reviewers and editors of journals and government funding committees (Abir-Am, 1992, p. 166). While in some cases proponents promoted institutionalization in a “concerted effort” to establish a new disciplinary paradigm addressing problems classical genetics “felt inadequate to solve” (Politi, 2018), in most cases molecular approaches and model organisms such as bacteria and phages seem to have been imported by smaller specialized subgroups or institutions with locally demarcated investigative pathways in mind.

Angela Creager shows, for instance, how Wendell Stanley's biochemical laboratory in Berkeley gradually shifted to viruses and new molecular approaches, in order to mobilize local funding for problems such as crop loss (Creager, 1996). Japanese biochemists, as Obayashi illustrates, pragmatically decided to incorporate certain molecular techniques with the goal to “change the methodology in [local] biochemistry” and despite that they did not understand the “original methodology and philosophical arguments of molecular biology” (Obayashi, 1986, p. 254). In Argentina, molecular approaches were narrated as potentially useful for vaccine production, which led to a range of “classical” geneticists and government funders to argue for their integration (Kreimer & Lugones, 2003, p. 48).^28^ Even at Cambridge, molecular genetics was only institutionalized as an independent discipline in 1962, after protracted negotiations with hesitant university administrators and multiple alternative attempts to integrate Crick in the classical genetics department had failed (Aicardi, 2016; Chadarevian, 1996, pp. 382–384; de Chadarevian, 2002).

These examples highlight, on the one hand, that there were a multitude of local alternative pathways in which molecular approaches could be integrated within existing disciplinary investigative pathways. On the other hand, they suggest that the subsequent institutionalization of molecular genetics as a discipline in its own right cannot be accounted for solely by the import of molecular approaches into existing experimental systems, as has been suggested by prominent historians of molecular biology such as Hans-Jörg Rheinberger (Rheinberger, 2009). Rather, these local cases of transfer of molecular techniques can be regarded as laying the “groundwork for a more visible institutional development that began in 1960” (Sloan, 2014). In other words, as evidence of the loosening of structural constraints characteristic of a juncture moment.

This raises the question on what the remarkably sudden and global institutionalization of molecular biology (Strasser, 2002, p. 516) and the demise of classical genetics as the dominant paradigm in biology was contingent upon. What propelled the rapid shift, within only a few years, from a comparatively “broad” definition of molecular approaches, often promoted as an “impetus” for classical genetics, biochemistry, and biophysics, toward a “narrow” disciplinary trajectory defined by a select set of problems, techniques, and objects (Olby, 1989 via Strasser, 2002)?

Based on my case study and the analysis of the secondary literature through the framework of narratives of contingency, I argue that this remarkably quick and broad takeover of molecular approaches and model objects in the discipline of genetics was contingent upon a sudden shift in how decision-makers narrated the future of biology. In the contingency/inevitability debate, the relevant point is that integration options still treated as viable in the late 1950s were rapidly selected out in the early 1960s, in a way that was causally sensitive to how actors reevaluated what was the most likely future direction. By narrating actual, but even more so contingent institutionalization and governmental support programs for molecular genetics elsewhere, the comparatively small group of proponents for a takeover of genetics managed to “convey a sense of the inevitability” of the direction of genetics and create a “sense of urgency” among various decision-makers to “catch up” (de Cock & Reinhardt, 2024, p. 126; Strasser, 2002, pp. 528–529). Programs by proponents of molecular approaches were now suddenly adapted without hesitance and significant national funding was directed towards new laboratory buildings, the acquisition of technical systems, the expansion of research staff and establishment of graduate and undergraduate training programs (Strasser, 2002). This simultaneously demanded a swift process of demarcation and decision-makers opted to appoint directors for the few investigative pathways that they, based on temporal and spatial comparisons with events elsewhere (de Cock & Reinhardt, 2024; Powell et al., 2007; Strasser, 2002, pp. 527–528), narrated to be most likely to define the new discipline trajectory.

This argument for a rapidly cascading “molecular revolution” in genetics that led to the contingent marginalization of classical mammalian genetics aligns with Politi's suggestion that scientific revolutions such as the formation of molecular biology can be approached as a “type of social change” at the level of local scientific communities (Politi, 2018). Read through narratives of contingency, the evidential payoff is that this “social change” is not only visible in later outcomes, but in how key actors in the moment narrated what counted as a viable future for their community and how their collective decisions would shape it. As my case study of the local skirmishes in Germany shows, this dynamic was evident in how a small group of decision-makers framed their pathway decisions as bearing on the future integrity of genetics as a community and discipline. Thus, Georg Melchers, who played a prominent role in the MPS debates and as chief editor of the country’s leading genetics journal promoted the integration of molecular approaches in order to preserve the “the unity of genetics”.^29^ Melchers was far from alone: several senior decision-makers framed their choices in terms of avoiding a partition, or even an end, of their community.

By the mid-1960s, proponents of a molecular takeover of genetics had begun to push a narrative in which the molecular takeover was not only desirable, but also inevitable. For example, Gunther Stent, initially appointed by Melchers exclusively to review phage genetics submissions in the journal, pushed for a complete shift toward molecular approaches, which he defined primarily as the “antithesis” to classical genetics. He consistently drew upon “historical examples” to argue that molecular genetics was “playing the role of biochemistry” by replacing classical genetics just as physiology had been displaced. By 1966, Stent observed that he had “decided to immolate myself” by forcing through a “separation” in both the journal and “the teaching program of Berkeley” by changing genetics courses to “end, rather than begin, with Mendel”.^30^

Revealingly of the sentiment of these pathway-discussions, this made Melchers compare Stent to the “extremists of the French and Russian revolutions,” while himself and other proponents of integration represented the “Girondist against the Jacobins or a Menshevik against the Bolsheviks”. As Melchers announced to Springer publisher Götze his “capitulation” to the separation of molecular and classical genetics, he remarked that he would undergo the “fate of someone who started a revolution with serenity” and that the “revolution in the journal had devoured its own children”.^31^ Read through the framework of narratives of contingency, these exchanges reveal a mechanism of selection-out: the integration narrative that had appeared most viable and supported in the late 1950s suddenly seen as out of step with the field’s direction. Some classical geneticists withdrew to “make place for the young activists”; and many decision-makers who had been hesitant or opposed a complete shift only a few years earlier acted strategically now that the new trajectory for genetics seemed settled.

In the context of supplying historical evidence for arguments in the philosophical-normative contingency/inevitability (C/I) debate, the preceding analysis has aimed to show how the narratives of contingency framework allows historians to organize case material so that it satisfies explicit evidential constraints. The historiography of molecular genetics is a particularly fertile arena for this, because it is broad, international, and richly institutional, offering comparable episodes across sites and decision-making settings. By making explicit when and why actors treated a situation as a juncture, how specific decisions turned into fork moments, which alternatives they articulated as viable, and which anticipated future conditions they mobilized to justify binding choices, such case studies become historically grounded points of departure for normative philosophical arguments about contingency and alternative pathway possibility. Had molecular techniques and model organisms been integrated locally within existing disciplines rather than forming a separate discipline, as many key decision-makers in genetics, biophysics, and biochemistry advocated in the 1950s, would this alternative trajectory have led to a fundamentally different developmental trajectory for the life sciences? Given the technical limitations of molecular techniques in the early 1960s for handling complex mammalian and human genetics, would such early integration have inevitably led to scientific dead-ends, ultimately forcing a return to “simpler” model organisms regardless of initial institutional choices? And were there, during the contingent molecular revolution in the mid-1960s, alternate local pathways possible that would have led to a different disciplinary formation of molecular genetics, characterized by other problems and model objects, as has been implied by Doris Zallen for photosynthesis and recently by Soraya de Chadarevian for cytogenetics (de Chadarevian, 2020; Zallen, 1993)?

## Conclusion: historical evidence and advancing the C/I problem into a productive and potentially interdisciplinary debate

This article has outlined how narratives of contingency can be advanced as a historical framework for producing and organizing evidence that can serve as a starting point for normative philosophical arguments of contingency and alternative pathway possibility in the development of science. It has shown how this framework satisfies three constraints that were identified as necessary for a historical case study to be viable as evidence for such arguments. First, narratives of contingency render critical junctures visible and highlight the various developmental directions that historical decision-makers considered. Second, it provides a historically grounded way of reconstructing explanatory factors for how and why specific pathways were put into motion while others were ignored or actively disregarded. Third, it facilitates an explicit delineation of the level and scale on which possible alternative pathways could be initiated, and a method to engage systematically with secondary literature in order to substantiate broader claims of contingency and alternative pathway possibility.

This type of systematic and explicit historical evidence can, in turn, help advance the C/I problem into a debate that is “productive” because it advances philosophical understanding of directionality in the development of science (Dellsén et al., 2024). I argue that it enables this in five concrete ways.

First, it allows philosophical arguments to move beyond the obstructive “success criterion” (Aylward, 2019), by taking as starting point historical evidence of a juncture moment where various pathways plausibly could have been put into motion. From this foundation, arguments for either contingency or inevitability no longer revolve around projecting speculative long-term triumphs or judging plausibility against ever-expanding present-day norms. Instead, the focus shifts to the epistemic, political, institutional, technological and material constraints that were historically present, and to how these constraints could plausibly shape the further development of an alternative pathway, or, conversely, contribute to its premature termination or decline into unproductive irrelevance.

Second, with this shared empirical foundation in historical case studies, philosophical debates can shift towards more explicit discussions about the epistemic significance of local alternative pathways. This would contribute to Hasok Chang’s call for attention to “lost pathways” in the development of science: pathways that may have been actively or accidentally neglected, prematurely “shut down,” and forgotten because of the “monistic and hegemonic tendencies” that characterize scientific progress, but that are still (or again) relevant today (Chang, 2021, p. 108). By grounding debate in such concrete historical examples of pathways that were dismissed or actively abandoned for a variety of identifiable factors, it can extend to the potential impact of these alternatives on contemporary organization of knowledge production.

Third, historical cases of local pathway decision-making processes enable philosophical arguments to focus on the specific factors upon which the enactment of scientific pathways is contingent. The narratives of contingency framework provides a way to connect a local case systematically to a wider historiography of a scientific episode, enabling debates about contingency to focus on identifiable mechanisms and conditions that influence directionality.

Fourth, this framework provides a concrete avenue for integrating history and philosophy of science (HPS) and, in this case, the history and philosophy of the life sciences—around a shared topic of interest: contingent directionality in the development of science. This framework could contribute to making the tensions of Kuhn’s legacy in philosophy and history of science productive by connecting, on the one hand, normative questions about paradigmatic patterns of change, and on the other hand, attention to local shifts in meso-level organizational structures.

Finally, I would argue for the urgency of revitalizing and broadening the C/I debate not only because it offers a promising avenue for HPS integration, but because it provides a way to enhance public, scientific, and policy understanding of how scientific knowledge is produced and how disciplinary directions are stabilized. The core questions of the C/I debate, why and how science develops as it does, and whether alternative directions are fundamentally possible, have proven remarkably engaging since Hacking’s initial formulation in the 1990s, and they are likely to attract broader attention once pursued systematically and explicitly in an interdisciplinary debate. While science undoubtedly succeeds partly because it describes and explains natural reality more effectively or accurately, the early stages of new scientific directions often depend on other factors, including institutional decision-making by small groups of stakeholders and situated judgments shaped by past experience and anticipated future conditions such as funding. Explaining where, how, and why scientific pathways were enacted, abandoned, or overlooked is therefore directly relevant for contemporary science policy and for public understanding of how and why the life sciences develop, or cease to develop, in particular directions.

## Data Availability

Photos of the used archive material can be provided upon request of the author or by visiting the Max Planck Society Archives in Berlin.

## Footnotes

1 Veli Virmajoki defines historical frameworks as explicit "sets of commitments" about how the world works that enable historiographical explanations. Adrian Currie and Kirsten Walsh observe that there is no "single correct framework that can be applied to a given scientific episode" as a framework’s purpose is to "generate and advance, rather than frustrate, historical and philosophical inquiry".

2 This takes also inspiration from philosophers of biology like McGhee who have argued that “evolution can be directional without being teleological”, rejecting the dominant dichotomy between contingency and directionless versus directionality and inevitability. *Contingent directionality* denotes fork moments in which a decision carrying temporal and causal weight was selected from a finite set of historical plausible options available, as conceived by narratives of contingency.

3 Howard Sankey, for example, highlighted that claims of inevitability are usually built on references to a “mind-independent reality” as the “target of scientific practices” which, combined with the “self-correcting nature of science”, form the basis of “consensus building” and therefore inevitably lead to the convergence on one successful scientific theory. Positions on contingency, on the other hand, typically rely on what John Beatty characterized as claims of "contingent upon". Here, certain initial starting conditions are identified as being both contingent and causally dependent for the development towards certain (alternative) outcomes.

4 Towards the end of his article Bouterse also suggests himself that this is impossible noting that “whether such an alternative local optimum exists and whether it was a genuine historical possibility at some point, is not a question that can be answered by historians alone”, Bouterse, 33.

5 Loison describes critical presentism as “the elaboration of a reciprocal critical relation between scientific knowledges from different times. Its legitimacy comes from the acknowledgement of both the contingency and the rationality of science.”

6 Kinzel and Jouni-Matti Kuukkanen have rightly criticized the implicit ways in which historians of science construct narratives by selecting and, more importantly, emphasizing and creating emplotments.

7 Martin and Bolinska have rightly observed that even when historians only implicitly emphasize certain factors as explanatory, they simultaneously claim their (relative) causal relevance in determining a series of events or trajectories and thus assert contingency.

8 These constraints are not intended as general historiographic requirements, nor as necessary conditions for historical explanation as such. Rather, they specify the conditions under which historical case studies can function as evidence for normative philosophical arguments about contingency and alternative pathway possibility.

9 Building on Steinmetz's critical engagement with Sewell's definition of historical events, I propose that critical junctures require two elements: an identification of lessened structural constraints (what Hillel terms "permissive and productive conditions") and historical actors' recognition that their actions could create significant change. Drawing on Poskett's recent conceptualization of scale as both actor and analytical category, this definition distinguishes critical junctures from structural transformation.

10 As Galison observed, in practice these constraints often intertwine, thus blurring “the boundary between the old categories of internal and external”.

11 As Carla Nappi has argued, the spatial level of analysis is simultaneously defined by the retrospective question-setting of a case study and by how actors employed these categories.

12 Remarkably broadly because Radick's case study is primarily historical in nature, yet his argument for contingency and alternative pathway possibility is both largely accepted by philosophers of science and his argument and evidence is discussed by practicing biologists and has been covered positively in prominent biology journals like Trends in Genetics and the FASEB Journal.

13 Radick also hints towards the importance of temporal comparisons, noting that both Weldon and Bateson relied heavily on the “use of the scientific past in trying to shape the present and future of the post-1900 science of heredity” (Disputed Inheritance 292).

14 Götze to Melchers 23.11.1959, AMPG, III. Abt., Rep. 75 Nr. 47.

15 Lehman to Hahn 01.09.1959, AMPG, II. Abt., Rep. 062 Nr. 0979.

16 Warburg Butenandt 04.04.1960, AMPG, III. Abt., Rep. 84 Nr. 7678.

17 Lehman to Hahn 01.09.1959, AMPG, III. Abt., Rep. 062. Nr. 0979.

18 Lynen Lehman 03.10.1959, AMPG, III. Abt., Rep. 062. Nr. 0979.

19 Lynen Lehman 03.10.1959, AMPG, III. Abt., Rep. 062. Nr. 0979.

20 Lynen Lehman 03.10.1959, AMPG, III. Abt., Rep. 062. Nr. 0979.

21 and protocol of the MPS Section for Biological-Medical Sciences, Lehman to Feodor Lynen 05.10.1959, AMPG, II. Abt., Rep. 62, Nr. 979.

22 Lehman to Feodor Lynen 05.10.1959, AMPG, II. Abt., Rep. 62, Nr. 979.

23 Karl Thomas to Ballreich 12.05.1961, AMPG II. Abt., Rep. 66, Nr. 1131.

24 Lehmann to Lynen, 05.10.1959, AMPG, II. Abt., Rep. 62, Nr. 979. Minutes of the Commission 14.11.1962, AMPG II. Abt., Rep. 66, Nr. 1131, Lehmann to Hahn 01.08.1959, AMPG, II. Abt., Rep. 062 Nr. 0979.

25 Hahn to Melchers 15.06.1959, Melchers to Hahn 20.06.1959, AMPG III. Abt., Rep. 14, Nr. 2869.

26 Lehman to Hahn 01.09.1959, AMPG, II. Abt., Rep. 062 Nr. 0979.

27 As Charlotte Auerbach argued in the closely related pathway decision-making debates about the appointment of Kaudewitz as editor in the journal at the time. Auerbach to Melchers 02.08.1960, AMPG, III. Abt., Rep. 75 Nr. 47.

28 As Kreimer and Lugones observe: “even if scientists did not realize that molecular biology actually represented an innovation, they tended to favor new research techniques that broadened without disrupting ‘traditional’ biology”.

29 Stubbe to Melchers 15.08.1966, AMPG, III. Abt., Rep. 75 Nr. 51.

30 Stent to Springer and Melchers, 10.06.1966, AMPG, III. Abt., Rep. 75 Nr. 51.

31 Melchers to Götze 05.07.1966, and Götze to Melchers 08.07.1966, AMPG, III. Abt., Rep. 75 Nr. 51.
